# Late Bronze Age climate change and the destruction of the Mycenaean Palace of Nestor at Pylos

**DOI:** 10.1371/journal.pone.0189447

**Published:** 2017-12-27

**Authors:** Martin Finné, Karin Holmgren, Chuan-Chou Shen, Hsun-Ming Hu, Meighan Boyd, Sharon Stocker

**Affiliations:** 1 Department of Archaeology and Ancient History, Uppsala University, Uppsala, Sweden; 2 Department of Physical Geography, Stockholm University, Stockholm, Sweden; 3 Navarino Environmental Observatory, Navarino Dunes, Costa Navarino, Messinia, Greece; 4 Swedish University of Agricultural Sciences, Uppsala, Sweden; 5 High-Precision Mass Spectrometry and Environment Change Laboratory (HISPEC), Department of Geosciences, National Taiwan University, Taipei, Taiwan ROC; 6 Department of Earth Sciences, Royal Holloway, University of London, Egham, Surrey, United Kingdom; 7 Department of Classics, University of Cincinnati, 410 Blegen Library, Cincinnati, OH, United States of America; New York State Museum, UNITED STATES

## Abstract

This paper offers new high-resolution oxygen and carbon isotope data from Stalagmite S1 from Mavri Trypa Cave, SW Peloponnese. Our data provide the climate background to the destruction of the nearby Mycenaean Palace of Nestor at Pylos at the transition from Late Helladic (LH) IIIB to LH IIIC, ~3150–3130 years before present (before AD 1950, hereafter yrs BP) and the subsequent period. S1 is dated by 24 U-Th dates with an averaged precision of ±26 yrs (2σ), providing one of the most robust paleoclimate records from the eastern Mediterranean for the end of the Late Bronze Age (LBA). The δ^18^O record shows generally wetter conditions at the time when the Palace of Nestor at Pylos was destroyed, but a brief period of drier conditions around 3200 yrs BP may have disrupted the Mycenaean agricultural system that at the time was likely operating close to its limit. Gradually developing aridity after 3150 yrs BP, i.e. subsequent to the destruction, probably reduced crop yields and helped to erode the basis for the reinstitution of a central authority and the Palace itself.

## 1. Introduction

The impact of past climate variability and abrupt climate change on ancient human societies is an ongoing debate. This debate often focuses around 1) certain time periods, commonly around so-called climate events, i.e. times when the climate rapidly changed, e.g. at 8200, 4200 and 3200 years before present (before AD 1950, hereafter yrs BP), and 2) archaeologically rich areas, such as SE Mexico (Yucatan Peninsula), the Indus Valley and the eastern Mediterranean [1–6]. In the eastern Mediterranean, there has been intense discussion about the impact of climate change on the fall of the Akkadian Empire and the end of the Late Bronze Age (LBA) occurring at ~4200 and ~3200 yrs BP respectively [7–18].

The debate about the causes of the rapid demise of many societies in the eastern Mediterranean at the end of the LBA includes a number of factors such as climate change, earthquakes, famine, political instability and/or invasions by the infamous Sea Peoples [17,19–23]. Lately the number of studies investigating the role played by climate at the end of the LBA in the eastern Mediterranean has increased and a number of them suggest aridity as a major factor [9,10,12,14,15,18]. Paleoclimate data, primarily from Cyprus and the Levant, suggest that a 300-year period of arid conditions that began around 3200 yrs BP led to reductions in agricultural productivity and subsequently contributed to a general socioeconomic crisis in the eastern Mediterranean [10,14,15,18]. However, it was recently pointed out that many of these datasets do not have sufficient chronological resolution to reliably tie climate information to archaeological data [20]. Despite chronological uncertainties and the fact that attributing sociopolitical changes to drought can be seen as overly simplistic and deterministic [21,24,25], the idea of widespread aridity has recently gained a prominent position in discussions about LBA societal change in the eastern Mediterranean as well as on the Greek mainland. Even though direct climate evidence from mainland Greece has not been available for this period, it has been suggested that the destruction of the Mycenaean Palatial centers towards the end of the LBA should be viewed in light of the severe aridity recorded for this period elsewhere see e.g. [10]. In this paper, we present new high-resolution paleoclimate data extracted from a cave located just off the coast of the Greek mainland, in close proximity to one of the major Mycenaean Palatial centers.

During the LBA the Mycenaean culture made a strong imprint on a large part of the Aegean world and in particular on the Peloponnesian Peninsula in southern Greece, where a number of palaces functioned as administrative and economic centers [26,27]. One of these was the Mycenaean Palace of Nestor at Pylos, located in present day Messinia in the SW Peloponnese, which controlled large areas of land in that region. The Mycenaean culture reached its zenith between approximately 3350 and 3150 yrs BP. The destruction of the Mycenaean palaces throughout the Peloponnese occurred at the transition between the Late Helladic (LH) IIIB (~3280 to ~3150 yrs BP) and LH IIIC (~3150 to ~3020 yrs BP). It was followed by a period of abrupt decline, during which the use of writing (Linear B) and complex forms of political, economic and social organization disappeared, settlement patterns changed, and the size and number of sites were drastically reduced [26–32]. However, some Mycenaean cultural traits continued throughout the LH IIIC period [26,29]. In addition, there is also evidence for rebuilding at the Mycenaean palace at Tiryns, some 120 km to the NE of Pylos [33]. By the Protogeometric period (~3020 yrs BP), new social frameworks were established displacing Mycenaean traditions [26,29]. The destruction of the palaces has often been seen as the prime marker for societal change on the mainland. However, the broader question is not why the palaces were destroyed, but rather why they were not rebuilt [12]. Several factors, among them rapid climate change, have been discussed as triggers for the sudden destruction of the palaces and the inability of Mycenaean society to recover [26,29,30].

The Mycenaean Palace of Nestor at Pylos provides a rare case study where textual and archaeological evidence can be combined to offer a more complete picture of the local economy (**S1 File**). The Palace functioned as the central administrative center of ancient Messinia and played a crucial role in the economy, but it did not maintain control over every aspect [34,35]. Mycenaean palaces were redistributive centers, although in a more nuanced manner than traditionally thought, based upon their ability to mobilize resources [36–38]. The economy was organized around the production, acquisition and distribution of raw materials and prestige goods, such as textiles, i.e., linen and wool, and perfumed olive oil [34,35]. The Palace did not, however, maintain control over the production of all staple goods; for example Linear B tablets indicate that there were local, independent small-scale producers of cereal crops [34,35]. The agricultural economy of the area was largely rainfed and land use would have ranged from large-scale flax fields and olive groves, possibly grazed by sheep, to small-scale units of land devoted to cereal production [34,35]. The importance of land in the Pylian economy is evident from textual evidence, much of which is concerned with landholdings and crops, and some of the land near the Palace is measured in seed-grain [36,38]. Although the Mycenaeans were familiar with hydrological engineering, there was a strong dependence on winter and spring precipitation for crop yields, a dependence that carries through to modern times [39,40].

Throughout the LH IIIB period the increase in the size and number of sites in the area surrounding the Palace is a good indicator of population growth in Messinia (**S1 File**). Such a demographic increase likely added additional stresses to the agricultural system and forced the cultivation of marginal lands. Agricultural products sustained the majority of the population of the kingdom of Pylos, and a dynamic relationship existed between the agricultural and social systems [41]. During the height of the LH IIIB period, the economy was a finely tuned system that depended on the proper functioning of all its components: agriculture, redistribution and tax collection [41].

The richness of the archaeological and textual evidence from the Peloponnese has, up to now, not been matched by the quality of the paleoclimate information from the area [42]. This means that inferences about socio-environmental links rely on paleoclimate data from other areas of the eastern Mediterranean, which, considering local variability, is problematic [11,42]. Here, we present a high-resolution, stable isotope based climate record from stalagmite S1 that formed from 4687 to 1297 yrs BP, in three discrete growth periods. The record covers large parts of the Greek Bronze Age, during which the Peloponnese saw the development of interconnected and complex societies and the intensification of agriculture, although with strong regional variability [43–45]. The record further covers the period extending from late Hellenistic times through the transition to the Byzantine period. The S1 proxy record sheds new light on the effects of climate on, the large scale social reorganization that occurred ~4200 yrs BP, the expansion in the area embraced by Mycenaean civilization ~3400 yrs BP, the subsequent destruction of the Mycenaean palaces ~3200 yrs BP, and the expansion in the number of rural settlements in the late Roman period. Here, however, we evaluate and investigate principally the chronological fit between variability in climate and the destruction of the Mycenaean Palace at Pylos and the subsequent sociopolitical change that took place at the end of the palatial period on the Greek mainland.

## 2. Methods and results

**Fig 1:**
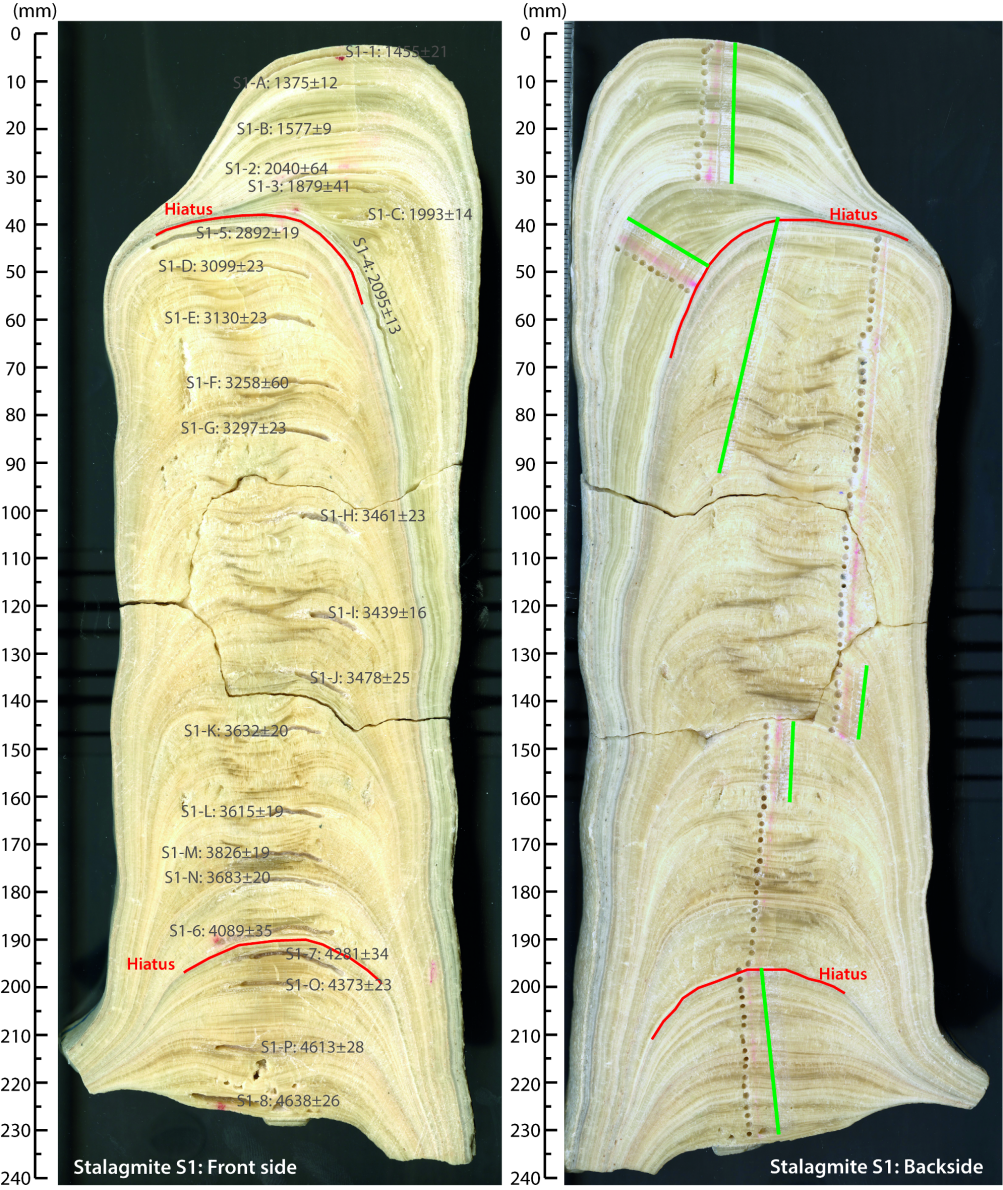
Image of stalagmite S1. Visible are the two sides of the central slab. Image on left shows the location of the samples milled for U-Th dating together with the results. Image on right shows holes from conventional drilling for samples for stable isotope analysis, together with tracks from sub-millimeter micromilling (highlighted by green lines). Red lines in both images indicate the position of inferred depositional hiatuses.

**Fig 2:**
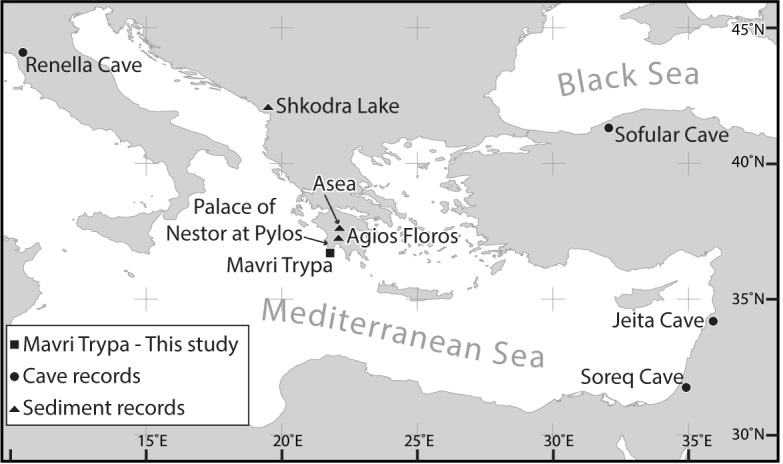
Location map. The location of Mavri Trypa Cave in relation to the Mycenaean Palace of Nestor at Pylos and other paleoclimate records mentioned in the text. Map made with Natural Earth.

Candle-shaped stalagmite S1, which is 230 mm in length (**Fig 1**), was collected in Mavri Trypa (Black Hole) Cave, located in the central part of Schiza Island, ~4 km off the SW coast of the Peloponnese (N36.7360° E21.7596°) (**Fig 2; S2 File and S3 File**). Permission for visiting and sampling the cave was issued by the Ephorate of Palaeoanthropology and Speleology, Athens, Greece. The cave formed in bedded Paleocene-Eocene limestones at 70 m above sea level. Twenty-four subsamples, 50–100 mg each, for U-Th dating [46,47] were milled along growth layers (**S3 File**). For carbon and oxygen stable isotopic measurements, subsamples were milled along a profile parallel to the central growth axis at 0.3-2-mm intervals (**S3 File**). Results are reported relative to the Vienna Pee Dee Belemnite (VPDB) standard.

**Fig 3:**
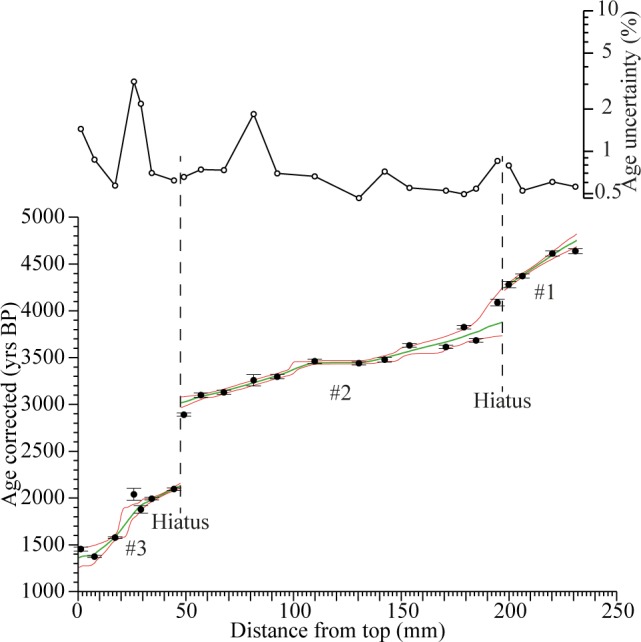
Age-depth model for S1 with uncertainties. Upper: Age uncertainties for each corrected U-Th age as percentages. Note logarithmic scale. Lower: Age-depth model for stalagmite S1 (green line) with corresponding 95% confidence limits (red lines). Black circles indicate individual U-Th ages, error bars show 2σ uncertainties. #1 to #3 denotes growth periods separated by hiatuses.

The corrected U-Th ages for S1 are precise with an average uncertainty <±1%. Twenty of 24 samples fall in stratigraphical order within uncertainties. High [^230^Th/^232^Th] values in all 24 samples indicate that only minimal age corrections are needed [48] (**Fig 3; S1 Table, S4 File**).

S1 mainly consists of open columnar calcite. Micrite and new crystal nucleation indicate growth interruptions at 47.7 and 197.5 mm from the stalagmite top (**S1 Fig, S4 File**). Large chronological shifts in adjacent U-Th samples at these levels indicate that these interruptions are associated with extended growth hiatuses. Age-depth modeling using *StalAge* [49] (v. 1.0) suggests that stalagmite S1 formed between 4687±68 and 1297±103 yrs BP in three individual growth periods: from 4687±68 to 4182±33 yrs BP (#1), from 3813±370 to 2953±63 yrs BP (#2), and from 2067±27 to 1297±103 yrs BP (#3) (**Fig 3; S4 File**).

δ^18^O and δ^13^C range from -3.74 to -5.99% and from -6.15 to -11.07%, respectively (**S2 Fig, S3 File, S1 Dataset**). There is a positive correlation between δ^18^O and δ^13^C along the growth axis in all three growth periods: #1 r = 0.80 (n = 113), #2 r = 0.84 (n = 146), and #3 r = 0.66 (n = 96), all significant at the 95% level.

## 3. Interpretation and discussion

Speleothem δ^18^O can, under certain conditions, be a good proxy for hydro-climatic change. Drip water δ^18^O, which reflects the isotopic composition of infiltrating meteoric water, in combination with processes occurring during percolation, controls speleothem δ^18^O [50]. A number of processes that relate to the climate-system control precipitation δ^18^O, including precipitation amount and seasonality, moisture source and transport distance, as well as condensation temperature [51]. Further processes in the atmosphere, such as the proportions of low-intensity stratiform vs high-intensity convective precipitation, altitudinal transportation of raindrops within rainfall systems, and evaporation below the cloud base, have also been shown to affect δ^18^O in precipitation, highlighting the complex nature of δ^18^O values in meteoric waters [52,53].

Previous studies of δ^18^O in speleothems from the eastern Mediterranean demonstrate that the precipitation amount often is a main control when sea surface conditions remain close to constant [54–66]. In the Peloponnese, precipitation δ^18^O values are more depleted during winter because of lower surface air temperature. However, in an annual cycle, there is a negative correlation between precipitation δ^18^O and rainfall amount, i.e. depletion increases with the precipitation amount, especially when the monthly average is below 100 mm [67]. A series of stable oxygen and hydrogen measurements on precipitation samples collected ~40 km north of Mavri Trypa Cave between January 2012 and September 2015 confirm the inverse relationship between precipitation amount and δ^18^O depletion [68]. A comparison between meteorological data and δ^18^O values from a modern stalagmite from Kapsia Cave in the central Peloponnese reveals a strong connection between δ^18^O depletion and an increase in the precipitation amount during the period of positive water excess (ONDJFMA) [69]. Considering the thickness of the bedrock above Mavri Trypa Cave, which is around 40 m, and that the catchment area for the karstic aquifer is relatively confined, given its location on an island, the climate signal from Mavri Trypa is not affected by aquifer mixing processes to a large extent. This could, however, also result in a relatively quick drying out of the aquifer under dry climate conditions. The two growth hiatuses and the inferred termination of deposition in Stalagmite S1 are likely a result of the aquifer drying out and they occur after longer periods of enriched δ^18^O and δ^13^C values.

Speleothem δ^13^C remains more complex to interpret than δ^18^O. Recent studies e.g. [70] have taken the interpretation beyond the influence of vegetation shifts from C_3_ to C_4_ [71,72] to a better understanding of the role of prior calcite precipitation (PCP) [73], the dead carbon component from the host rock, atmospheric CO_2_, the CO_2_ of the soil zone, and CO_2_ resulting from the decay of organic material found within the vadose zone [74] on speleothem δ^13^C. In the Mediterranean region and other semi-arid areas, depleted δ^13^C in speleothems has been linked to increased biological activity, including soil microbial activity, above the cave [54,56,75–77]. Biological activity, in turn, is linked to precipitation in many areas of the Mediterranean, including our study area. Thus positive covariation between δ^13^C values and hydro-climate variability, as recorded by δ^18^O, is to be expected unless human activities above the cave, such as grazing or deforestation, have affected the vegetation [54,56,64,75,78].

The strong correlation between δ^18^O and δ^13^C values in the three growth periods indicates that Stalagmite S1 did not form in isotopic equilibrium with its parent drip water. Few stalagmites form under true equilibrium conditions [79], especially in semi-arid environments, and kinetic effects should be considered [77,80]. Kinetic fractionation is mainly driven by evaporation of drip water emerging in the cave due to low relative humidity (RH) in the cave air, and by the degassing of CO_2_ from the parent water due to differences in CO_2_ partial pressure (pCO_2_) between water and cave air. During drier climate conditions, δ^18^O will be enriched by increased evaporation in the epikarst and in the cave due to low RH, and δ^13^C will be enriched by increased degassing connected to reduced drip rates [81], enhancing any signal of drought in the stalagmite. Additionally, PCP may enrich δ^13^C values and since this factor typically is enhanced during drier climate conditions, it also acts to enhance the enrichment signature of δ^13^C during more arid phases. In short, a strong positive correlation between δ^18^O and δ^13^C is expected since both proxies respond to similar environmental drivers and similarly to kinetic effects. In addition, the closed nature of the cave and the high RH in the cave air (**S2 File**) should act to significantly reduce the effects of kinetic fractionation.

Based on the above discussion we suggest that the δ^18^O in the stalagmite from Mavri Trypa Cave should be interpreted as a signal for moisture, although there may be an influence of kinetic fractionation, with more negative δ^18^O values indicating wetter conditions and vice versa. The δ^13^C signal from Mavri Trypa may tentatively be used as a proxy for biological activity above the cave linked to moisture availability. However, in this paper we favor the use of δ^18^O over δ^13^C for making interpretations about past hydro-climatic variability, although we use the δ^13^C signal to better understand the δ^18^O.

### 3.1 Climate during the Bronze Age and from the Hellenistic to the Byzantine period

The Mavri Trypa δ^18^O record is supported by other stable isotope records from the Mediterranean region indicating a large-scale control on the isotopic signal [58,65,75,76,82–85] (**S3 Fig**).

**Fig 4:**
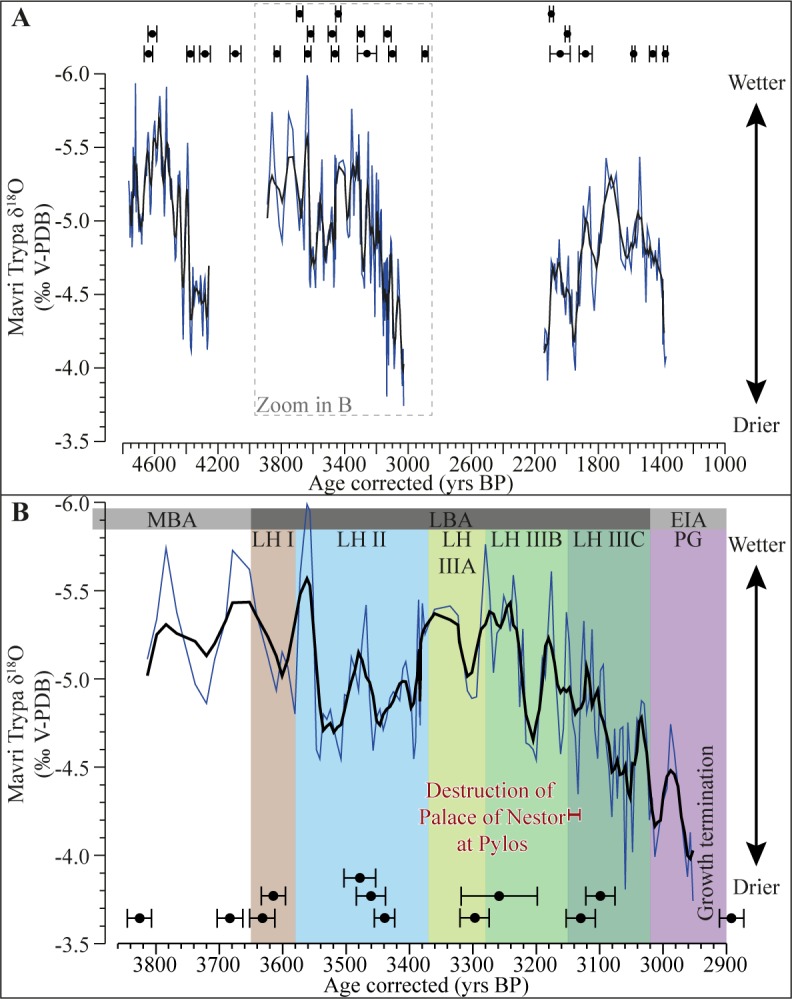
Stable oxygen isotopes as a proxy for precipitation showing wetter and drier periods through time. A: Full sequence of stable oxygen isotopes from S1 plotted against time. Individual U-Th ages presented as black circles, error bars show 2σ uncertainties. B: Stable oxygen isotope results covering the Late Bronze Age (LBA) and Early Iron Age (EIA). The sub-periods of the LBA (Late Helladic (LH) I to LH IIIC) and the Protogeometric (PG) are shown together with the suggested period when the Palace of Nestor at Pylos was destroyed based on information from [27,29,31].

The δ^18^O and δ^13^C results from Mavri Trypa indicate relatively wet conditions from 4700 yrs BP to 4500 yrs BP, followed by a transitional period with large isotopic fluctuations from 4500 yrs BP to 4300 yrs BP leading towards drier conditions (**Fig 4; S2 Fig**). A diatom record from the nearby Agios Floros wetland also shows a development of aridity from 4500 yrs BP [86]. Stable arid conditions in Mavri Trypa occur from 4300 yrs BP until stalagmite S1 stops growing at 4200 yrs BP. There is widespread evidence from the eastern Mediterranean region in general for more arid conditions around 4200 yrs BP [42,58].

Growth in stalagmite S1 resumes around 3800 yrs BP with δ^18^O values indicating wetter conditions (**Fig 4**). The resumption concurs with a period of wetter conditions suggested by other proxy data from the Peloponnese [45]. The generally wetter conditions indicated by our record last until 3150 yrs BP, although interrupted by two periods of drier conditions. The first drier phase develops rapidly at 3550 yrs BP and lasts until 3400 yrs BP. This phase is roughly divided in half by a brief return to wetter conditions at 3450 yrs BP. The second dry phase also develops rapidly and is centered around 3200 yrs BP (**Fig 4**) (see extended discussion below). The drier periods are also characterized by enriched δ^13^C values interpreted as indicating reduced biological activity above the cave, supporting the δ^18^O signal (**S2 Fig**). In Mavri Trypa a transition towards drier conditions starts ~3150 yrs BP, marking the gradual end of the wetter period. The transitional period is one of high-amplitude isotopic fluctuations on a decadal scale, superimposed on a centennial scale trend towards less negative δ^18^O values, indicating reduced precipitation. Overall more arid conditions are evident from 3100 yrs BP until 2950 yrs BP when growth in S1 terminates again. At this time, there is regional and local evidence from the eastern and central Mediterranean for drier conditions e.g. [42,45]. High-resolution stable isotope records from Jeita Cave [65], Renella Cave [58], Sofular Cave [75,76], and Nar Gölü [85] all show that aridity starts to develop around 3200 years BP but that the most arid conditions only occur after 3200 yrs BP (**S3 Fig**). From a chronological perspective, these records are some of the most robust paleoclimate records for the period around 3200 yrs BP (**S4 Fig**).

The third growth period in the Mavri Trypa stalagmite starts at 2050 yrs BP and coincides with the Roman Warm Period. The overall enrichment in the δ^18^O values that occurred during the first 200 years of this period likely results from these samples being milled towards the flank of the stalagmite in this section and is not related to climate (**S2 Fig**). Other paleoclimate records from the Peloponnese indicate wetter conditions from ~2550 yrs BP [45,64,86,87]. It is possible that the duration of the second hiatus in the Mavri Trypa stalagmite is not only a result of drier conditions, but also relates to the rerouting of water through the bedrock or human intervention in or near the cave. Following a brief interval of arid conditions just before 1900 yrs BP, a wetter period between 1850 and 1300 yrs BP, which peaks at 1675 yrs BP, is evident (**Fig 4**). After 1300 yrs BP there is a rapid change to drier conditions and the stalagmite stops growing 1297±27 yrs BP. Around this time many records from the eastern and central Mediterranean indicate drier conditions, and speleothem formation also ceased in Kapsia Cave in the central Peloponnese and in nearby Alepotrypa Cave, possibly indicating both local and regional aridity (**S3 Fig**) [54,64,65,75,76,84,88].

### 3.2 Climate, the destruction of the Mycenaean Palace of Nestor at Pylos and the end of the Late Bronze Age

The sampling resolution in the Mavri Trypa record for the period 3350–3000 yrs BP (i.e. much of the LBA) is on average 5 years and the uncertainty in the age-depth model is on average ±31.5 years, making it one of the most precise paleoclimate records for the LBA in the eastern Mediterranean (**Fig 3**, **S4 Fig**). Thus, the record approaches the criteria Knapp and Manning [20] argue are necessary in order to reliably compare archaeological events to climate data. Considering the proximity between Mavri Trypa and the Palace of Nestor, the paleoclimate information from this cave is pertinent for evaluating the potential impact of climate on the destruction of that Mycenaean palace and social processes during the subsequent periods. The archaeological chronology around 3200 yrs BP in the Peloponnese is generally well accepted, although some uncertainties remain. It is based on the cross-dating of ceramic material with “fixed points” in Egyptian chronology and contextually secure radiocarbon dates [20,89–91]. We are thus able to rather precisely compare our paleoclimate data with the timing of the destruction of the Palace of Nestor and the end of the LBA in the Peloponnese.

The destruction of the Palace of Nestor is thought to have occurred ~3150–3130 yrs BP [27,29,31] (**Fig 4**). There is no unequivocal evidence for a prolonged period of drier conditions in the Mavri Trypa record at that time. Instead, it appears that the period around 3150 yrs BP is one of generally wetter, albeit fluctuating, climate conditions marking the beginning of a transitional period that leads into a distinct period of drier conditions that exists from ~3100 yrs BP (**Fig 4**). At the time of the destruction of the Palace there is a very short period of enriched δ^18^O values (mainly defined by one measurement point); this fluctuation, however, is not evident in the δ^13^C (**S2 Fig**). Considering the expected contribution of local noise to the δ^18^O signal and the lack of response in the δ^13^C, we suggest this enrichment cannot not be linked to drought, at least not of the magnitude seen around 3200 yrs BP and after 3100 yrs BP.

Before the Palace is destroyed, the Mavri Trypa record shows evidence for a drier period around 3200 yrs BP that lasted ~20 years. This dry period can be firmly placed in the LH IIIB period and, given the new data from Mavri Trypa, occurs, taking the age uncertainties in to account, two to eight decades before the palace is destroyed. This dry interval is followed by a return to wetter conditions, before the transitional period leading towards drier conditions that begins at 3150 yrs BP. It seems from the Mavri Trypa record that the dry phase around 3200 yrs BP was minor compared to what would come ~100 years later. The record from Mavri Trypa suggests that it was in the postpalatial period that arid conditions developed, and that it is only after the Bronze Age, in the Protogeometric period, that very arid conditions were established (**Fig 4).** The dry conditions recorded from 3100 yrs BP firmly belong to the LH IIIC period and likely contributed to the inability of the Mycenaean palaces in the Peloponnese to reassert their power.

Other paleoclimate records from the Peloponnese do not offer as much detail as the record from Mavri Trypa for the LH IIIB/LH IIIC transition [45], although a sedimentary record from the nearby Asea Valley indicates gradually cooling conditions from 3200 to 2700 yrs BP [87].

The stable isotope record from Mavri Trypa, together with other stable isotope records from the region, suggests that the end of the LBA (LH IIIC) is marked by increasing aridity but that it only reached its peak after ~3000 yrs BP.

Where does the new paleoclimate data from Mavri Trypa, with its current age-depth model and uncertainties, leave us in relation to the possible influence of climate on the destruction of the Mycenaean Palace of Nestor at Pylos and to the broader question of why it was not rebuilt in the LH IIIC period? The drought recorded at 3200 yrs BP clearly precedes the destruction of the Palace. Evidently the centralized administrative system controlled by the Palace could survive such a relatively short-term dry period and remain in control. Some fifty years later, however, when the pronounced period of aridity started to develop, the system would crumble. Further east, it has been proposed that triggers such as social unrest linked to drought induced food shortages could have been instrumental in LBA change [14,18] and there are few reasons to think that the mainly rainfed agricultural system of Messinia was less susceptible to those stresses. In this area, which naturally suffers from erratic rainfall, one of the principal ways in which the Palace central authority made themselves essential to small-scale producers was by providing food security in the case of crop failure or shortfalls during drought [34,39]. Although the record from Mavri Trypa does not offer any clear evidence for altered climate conditions that could have acted as a trigger for the destruction event, the period of drought around 3200 yrs BP could have contributed to the destabilization of the political and economic order. Increased aridity could have led to reduced agricultural output affecting the finely tuned economic system of a society that was close to, or already, over-extended, rattling the very foundations of the fragile palatial economy (**S5 File)**. Although the palatial society at Pylos survived the short-term drought around 3200 yrs BP, it may have destabilized, or at least challenged, the system, which produced archaeologically and textually discernible responses by the Mycenaean elite. The suggestion of social turbulence and larger scale socioeconomic problems is hinted at both in the Linear B tablets and other evidence from the Palace; storage was increased and access to the Palace was restricted shortly before the destruction (**S5 File**) [36,92]. The new climate evidence from the Greek mainland, while not directly supporting a climate explanation for the destruction of the Palace, suggests that drier local conditions was one of several factors contributing to its demise. Rather than viewing the evidence of climate change as a cause of the collapse, we view it as part of the process of destabilization that contributed to the palatial administration's inability to reconstruct social hierarchies after the destruction. It has been suggested that the largely synchronous abandonment of the palatial centers across the Peloponnese at the end of the LH IIIB was, rather than an event, a process that took decades, and a short-term downturn in climate can be seen as one of many drivers [12].

The new data from Mavri Trypa also provides an opportunity to investigate the climate backdrop to the question of why the Mycenaean elite did not re-form and why the Palace was not rebuilt. Many signs of political and social collapse are visible in Pylos and Messinia as a whole after the destruction of the Palace. For instance, there was no urban reconstruction or subsequent cultural regeneration on the acropolis or in the adjacent lower town at Pylos and a pronounced depopulation of Messinia is evident from survey results [36]. The clear trend toward aridity from 3150 yrs BP probably meant a gradual increase in the number of years of drought, leading to failed crops or strongly reduced yields, and, more importantly, that agricultural productivity in normal and good years was reduced [93]. This in turn meant that it became increasingly difficult for farmers in Messinia to produce a ‘normal surplus’ that could be stored and taxed, either directly or socially, which has been argued to be an important mechanism behind the creation of social elites [39]. In an environment with developing aridity and reduced crop yields it would have become increasingly difficult to produce the ‘natural surplus’ that would enable a central authority to reassert itself by providing food relief, or trigger the formation of new social elites. There is, however, evidence for the continuation of small-scale subsistence farming in Messinia in LH IIIC and through the Early Iron Age (EIA), albeit at a much reduced scale [34]. The new evidence from Mavri Trypa makes it possible for the first time to situate the trajectory of events following the destruction of the Mycenaean Palace of Nestor at Pylos within a period of developing aridity throughout LH IIIC and the early part of the EIA.

## 4. Conclusions

The influence of climate on the Mycenaean world and the destruction of the Palace of Nestor at Pylos can, for the first time, be assessed through the investigation of a local high-resolution δ^18^O record that is the most precisely dated paleoclimate record from the eastern Mediterranean for the end of the LBA.

The Mavri Trypa δ^18^O record shows little or no unequivocal evidence for drier conditions when the palace in Pylos is destroyed ~3150–3130 yrs BP, at the transition from LH IIIB to LH IIIC. While the new paleoclimate evidence from the Greek mainland does not support a clear chronological synchronism between the destruction of the Mycenaean Palace at Pylos and drier conditions, as has been suggested previously, it does offer an insight into difficulties that existed several decades before the collapse of the palatial system.

There is evidence for a dry phase extending for approximately two decades around 3200±30 yrs BP, which can be firmly placed in the LH IIIB period, i.e. before the destruction of the palace. Evidently the centralized administrative system at Pylos managed to survive that period of drought, although evidence suggests that cracks were beginning to emerge in the period immediately preceding the palace’s destruction. This dry period was slight in comparison to what would come 100 years later, both in terms of magnitude and duration, but it nevertheless would have been felt in the agriculturally dominant palace economy. With the evidence in hand, the precise reasons for the destruction should be sought beyond climate explanations, although the effect of the climate should be considered as a contributing factor.

Following the destruction of the Mycenaean Palace at Pylos, there is strong evidence that climate conditions became progressively more arid during the LH IIIC period, and pronounced aridity is evident at the very end of the LBA and in the Protogeometric period, before the Mavri Trypa stalagmite stopped growing at 2953±63 yrs BP. We suggest this clear trend towards drier conditions caused reduced agricultural output, hampering the restoration of a central authority or the formation of new social elites. Small-scale subsistence agriculture however, persisted in the area.

For the first time, there are indications that climate may be one mechanism behind the process that led to the failure of the Mycenaean way of life in Pylos and there is strong evidence that developing aridity following the destruction of the Palace made it difficult for social elites to re-form and for the palatial system to be re-established. One cannot, however, attribute the collapse of the Mycenaean way of life to a single monolithic cause or event. Instead one should look for a suite of factors that contributed to the inability of the palatial elite to reconstitute the political, economic, and social organization that existed at the end of LH IIIB. Climate change is certainly a critical component in the equation.

## Supporting information

S1 Fig Plate of petrographic thin sections from stalagmite S1. A-D show micrite and new crystal nucleation (red arrows) at 197.5 mm depth from the top indicating a growth interruption. A and B show the same slide in crossed polar light and plane-polarized light respectively. C and D show the same slide in crossed polar light and plane-polarized light respectively. E and F show new crystal nucleation at 47.7 mm depth from the top in crossed polar light and plane-polarized light respectively, indicating a growth interruption. At this depth, there is also an almost perpendicular change in the direction of the growth axis (see Fig 1). G shows a segment of the area between 55 and 145 mm depth from the top that has a more irregular and fibrous fabric toward the center compared to the flanks. Green arrows indicate direction of growth.
(TIF)

S2 Fig Stable oxygen (δ^18^O) and carbon (δ^13^C) isotopes from stalagmite S1 plotted vs. age. Thicker black line represents 5-point moving average. Note inverted y-axes.
(PDF)

S3 Fig Selected high-resolution stable isotope records from eastern and central Mediterranean compared the δ^18^O record from Mavri Trypa (this study). Bold black lines for each record represent a running average. The running average was selected for each individual record to filter the average resolution to ~30 years, the lowest resolution in any record in the figure, in order to enhance comparability between records. Wetter climate conditions are up and drier down. Records are organized from west to east. For the δ^18^O record from Soreq Cave no running average was calculated for the period of low resolution (i.e. between 3600 and 2000 yrs BP).
Similarly, colored bars indicate possible parallel periods of wetter and drier conditions in other records from central and eastern Mediterranean. Question marks indicate less certain matching with the record from Mavri Trypa.
(EPS)

S4 Fig Dating points and uncertainties from selected paleoclimate records from the central and eastern Mediterranean around 3200 yrs BP. Renella Cave [58]; Shkodra Lake [84]; Mavri Trypa (this study); Sofular Cave [75,76], and Jeita Cave [65]. The δ^18^O record from Nar Gölü [85] is not represented in this graph because the chronology is based on the counting of annual lamina. There is only one U-Th age (3770±310 [83]) from Soreq Cave within the span of the figure. Records are organized from west to east.
(EPS)

S5 Fig Comparison of δ^18^O and δ^13^C results from parallel tracks in stalagmite S1. Figure showing the similar isotopic signal in different but parallel tracks in stalagmite S1 indicating the stability of the signal. Slight offset on x-axis caused by imperfect matching between the results.
(TIF)

S1 Table U-Th dating chemistry and results table. Uranium and thorium isotopic compositions and ^230^Th ages for stalagmite S1 by MC-ICPMS, Thermo Electron Neptune, at HISPEC, NTU.
(DOCX)

S1 File Archaeological background to the Palace of Nestor at Pylos and the area around Pylos. (DOCX)

S2 File More detailed information about the setting of the cave where speleothem S1 was collected. (DOCX)

S3 File More detailed information about the material and methods. (DOCX)

S4 File More detailed information about the results of the analyses presented in this paper. (DOCX)

S5 File Extended discussion: Potential impacts of climate variability on Mycenaean society. (DOCX)

S1 Dataset Stable oxygen and carbon isotope data for stalagmite S1. (TXT)
