# Supplementary figures and images for: Late Bronze Age climate change and the destruction of the Mycenaean Palace of Nestor at Pylos
Source: PLoS One. 2017 Dec 27;12(12):e0189447. doi: 10.1371/journal.pone.0189447 (PMC5744937; doi:10.1371/journal.pone.0189447)

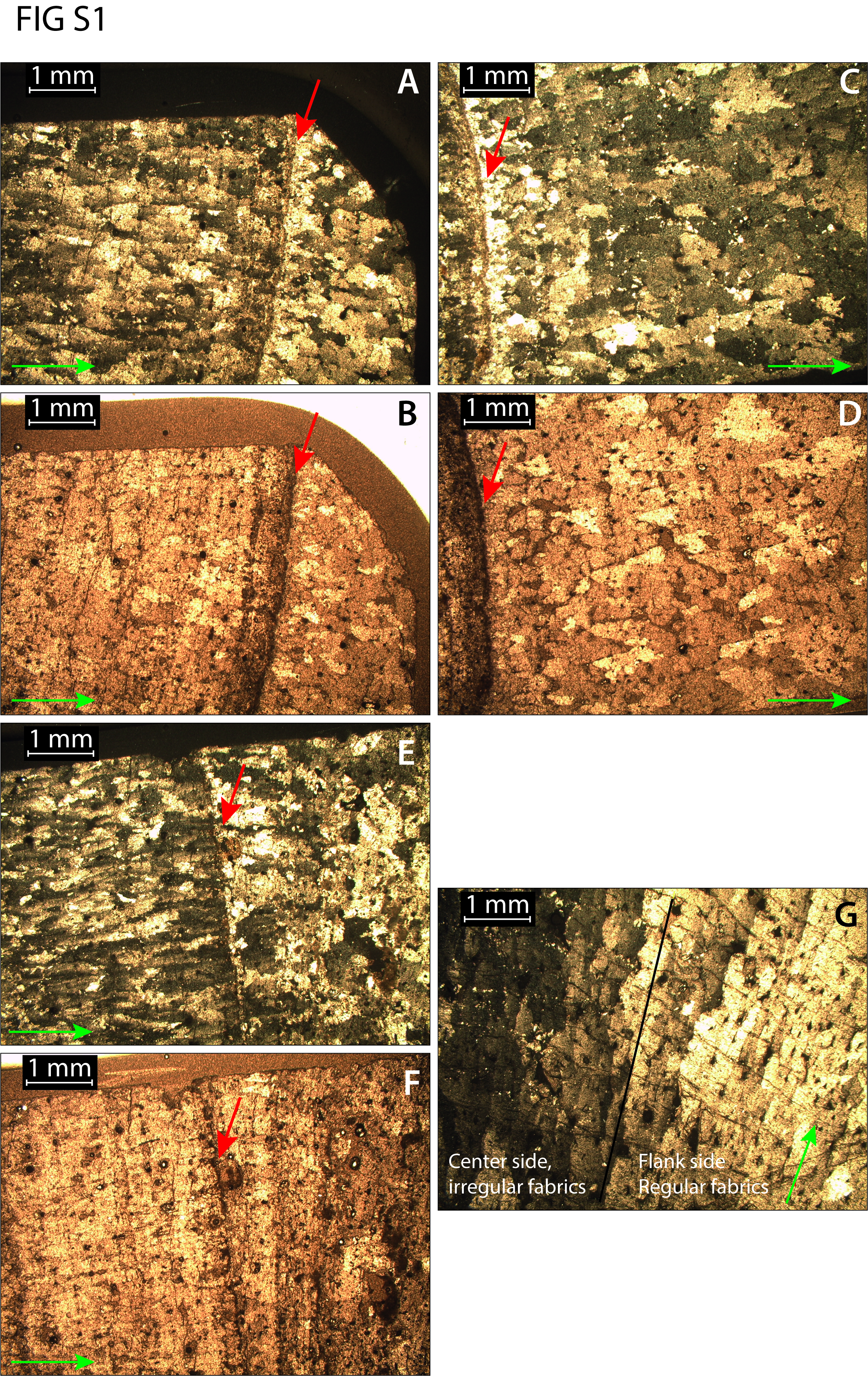

Supplement: S1 Fig — A-D show micrite and new crystal nucleation (red arrows) at 197.5 mm depth from the top indicating a growth interruption. A and B show the same slide in crossed polar light and plane-polarized light respectively. C and D show the same slide in crossed polar light and plane-polarized light respectively. E and F show new crystal nucleation at 47.7 mm depth from the top in crossed polar light and plane-polarized light respectively, indicating a growth interruption. At this depth, there is also an almost perpendicular change in the direction of the growth axis (see Fig 1). G shows a segment of the area between 55 and 145 mm depth from the top that has a more irregular and fibrous fabric toward the center compared to the flanks. Green arrows indicate direction of growth. (TIF) [file pone.0189447.s001.tif]

FIG S2

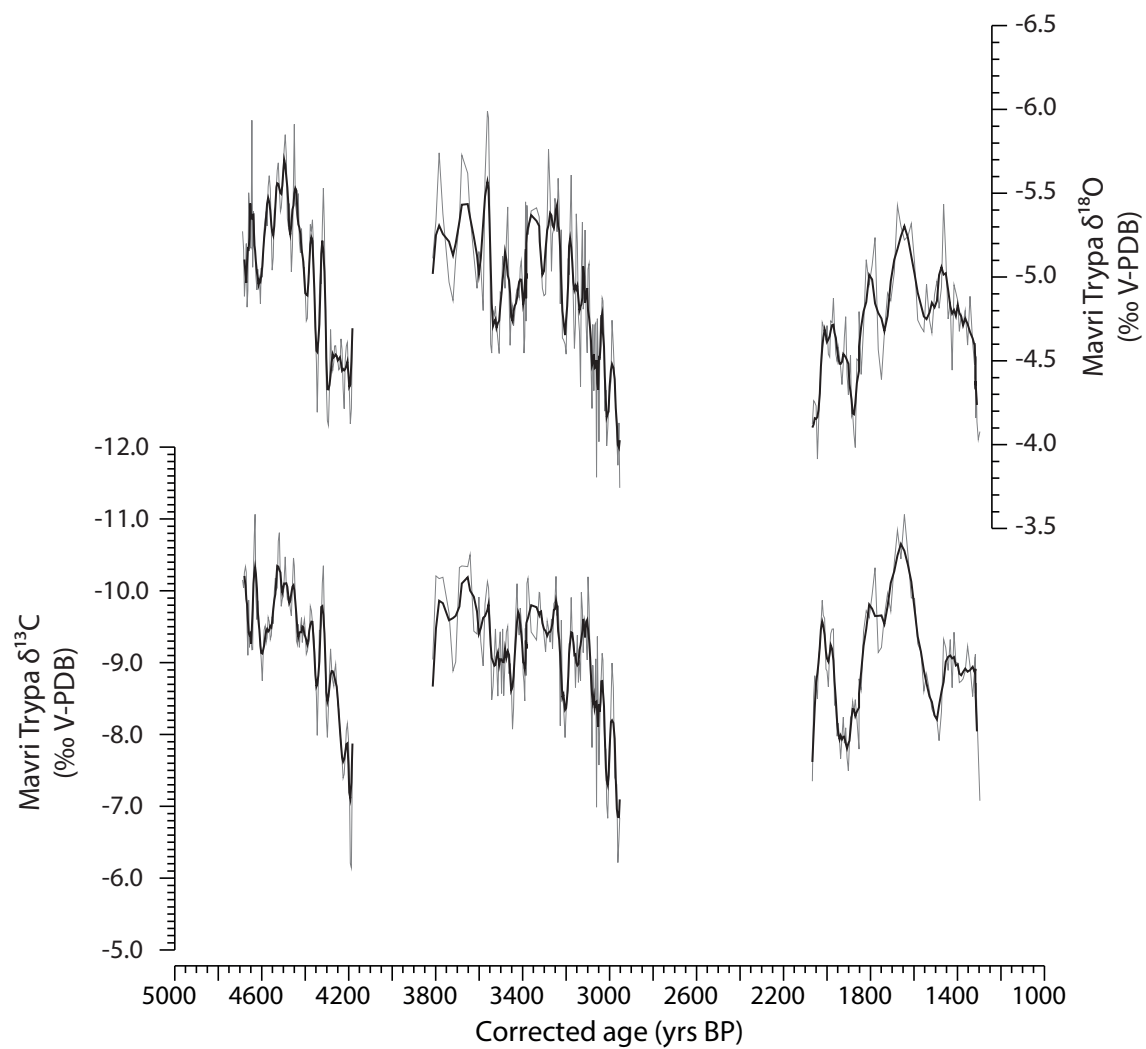

Supplement: S2 Fig — Thicker black line represents 5-point moving average. Note inverted y-axes. (PDF) [file pone.0189447.s002.pdf]

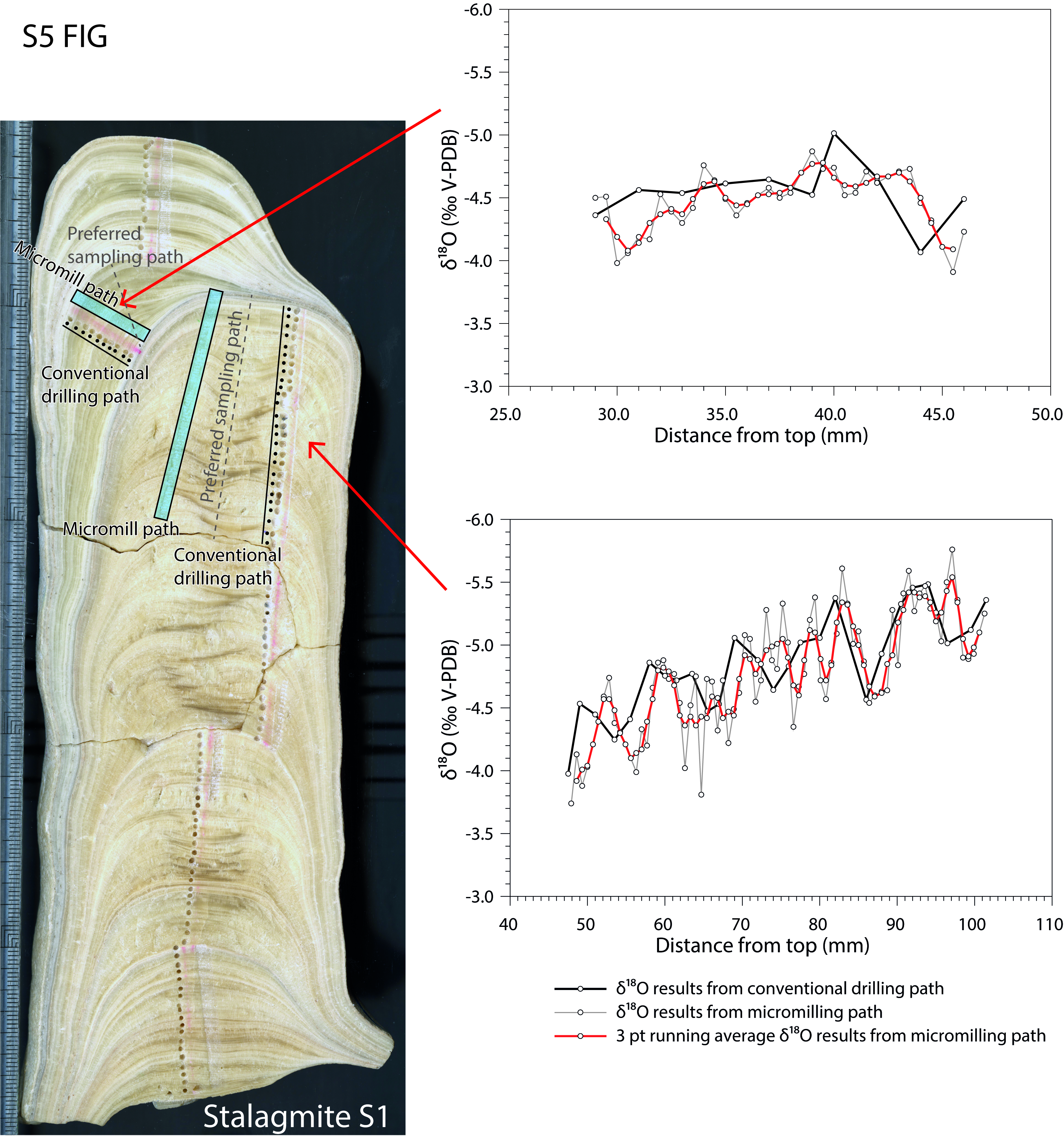

Supplement: S5 Fig — Figure showing the similar isotopic signal in different but parallel tracks in stalagmite S1 indicating the stability of the signal. Slight offset on x-axis caused by imperfect matching between the results. (TIF) [file pone.0189447.s005.tif]
