# Supplementary material for: Late Bronze Age climate change and the destruction of the Mycenaean Palace of Nestor at Pylos
Source: PLoS One. 2017 Dec 27;12(12):e0189447. doi: 10.1371/journal.pone.0189447 (PMC5744937; doi:10.1371/journal.pone.0189447)
Supplement: S1 Table — Uranium and thorium isotopic compositions and 230Th ages for stalagmite S1 by MC-ICPMS, Thermo Electron Neptune, at HISPEC, NTU. (DOCX) [file pone.0189447.s006.docx]

| **Sample**  **ID** | **Distance from top**  **(mm)** | **^238^U**  **10^-9^g/g***^a^* | | **^232^Th**  **10^-12^g/g** | | **δ^234^U**  **measured***^a^* | | **[^230^Th/^238^U]**  **activity***^c^* | | **^230^Th/^232^Th**  **atomic (x 10^-6^)** | | **Age (yr ago)**  **uncorrected** | | **Age (yr ago)**  **corrected *^c,d^*** | | **δ^234^U_initial_**  **corrected***^b^* | |
| --- | --- | --- | --- | --- | --- | --- | --- | --- | --- | --- | --- | --- | --- | --- | --- | --- | --- |
| **S1-1** | **1.2** | **1041.0** | **± 2.2** | **364.1** | **± 4.0** | **39.5** | **± 2.7** | **0.01386** | **± 0.00019** | **654** | **± 12** | **1 464** | **± 21** | **1 455** | **± 21** | **39.7** | **± 2.7** |
| **S1-A** | **7.6** | **901.46** | **± 0.92** | **54.3** | **± 7.6** | **38.1** | **± 1.2** | **0.01302** | **± 0.00011** | **3566** | **± 501** | **1 376** | **± 12** | **1 375** | **± 12** | **38.3** | **± 1.2** |
| **S1-B** | **17.1** | **991.8** | **± 1.2** | **28.7** | **± 4.0** | **31.3** | **± 1.4** | **0.014811** | **± 0.000084** | **8440** | **± 1187** | **1 577.3** | **± 9.2** | **1 576.5** | **± 9.2** | **31.4** | **± 1.4** |
| **S1-2** | **25.9** | **1098.6** | **± 2.1** | **280.1** | **± 4.4** | **17.0** | **± 2.3** | **0.01891** | **± 0.00058** | **1223** | **± 42** | **2 046** | **± 64** | **2 040** | **± 64** | **17.1** | **± 2.3** |
| **S1-3** | **29.1** | **1227.7** | **± 1.6** | **23.5** | **± 3.5** | **63.7** | **± 1.7** | **0.01818** | **± 0.00039** | **15689** | **± 2349** | **1 879** | **± 41** | **1 879** | **± 41** | **64.0** | **± 1.7** |
| **S1-C** | **34.2** | **1122.5** | **± 1.5** | **185.9** | **± 6.9** | **34.7** | **± 1.5** | **0.01879** | **± 0.00012** | **1871** | **± 71** | **1 998** | **± 13** | **1 993** | **± 14** | **34.9** | **± 1.5** |
| **S1-4** | **44.5** | **1352.9** | **± 1.6** | **57.8** | **± 4.5** | **35.5** | **± 1.6** | **0.01972** | **± 0.00012** | **7613** | **± 589** | **2 097** | **± 13** | **2 095** | **± 13** | **35.7** | **± 1.6** |
|  |  |  |  |  |  |  |  |  |  |  |  |  |  |  |  |  |  |
| **S1-5** | **49.1** | **850.2** | **± 1.2** | **358.8** | **± 3.3** | **31.6** | **± 2.6** | **0.02710** | **± 0.00015** | **1059** | **± 11** | **2 903** | **± 18** | **2 892** | **± 19** | **31.8** | **± 2.6** |
| **S1-D** | **57.0** | **880.4** | **± 1.2** | **207.9** | **± 8.3** | **39.0** | **± 1.6** | **0.02917** | **± 0.00021** | **2037** | **± 83** | **3 105** | **± 23** | **3 099** | **± 23** | **39.3** | **± 1.6** |
| **S1-E** | **67.7** | **889.3** | **± 1.3** | **149.2** | **± 5.9** | **41.6** | **± 1.6** | **0.02951** | **± 0.00021** | **2901** | **± 116** | **3 134** | **± 23** | **3 130** | **± 23** | **41.9** | **± 1.6** |
| **S1-F** | **81.5** | **799.9** | **± 1.1** | **122.3** | **± 5.9** | **38.2** | **± 1.6** | **0.03061** | **± 0.00055** | **3301** | **± 170** | **3 262** | **± 60** | **3 258** | **± 60** | **38.6** | **± 1.6** |
| **S1-G** | **92.5** | **718.0** | **± 1.1** | **390.0** | **± 6.1** | **37.3** | **± 1.6** | **0.03103** | **± 0.00020** | **942** | **± 16** | **3 311** | **± 22** | **3 297** | **± 23** | **37.7** | **± 1.6** |
| **S1-H** | **109.9** | **670.5** | **± 1.0** | **490.8** | **± 5.7** | **34.9** | **± 1.6** | **0.03250** | **± 0.00019** | **732.2** | **± 9.4** | **3 479** | **± 21** | **3 461** | **± 23** | **35.2** | **± 1.6** |
| **S1-I** | **130.4** | **657.61** | **± 0.67** | **202.5** | **± 5.3** | **34.1** | **± 1.4** | **0.03218** | **± 0.00014** | **1723** | **± 46** | **3 447** | **± 16** | **3 439** | **± 16** | **34.4** | **± 1.4** |
| **S1-J** | **142.3** | **842.0** | **± 1.0** | **178.7** | **± 7.0** | **36.7** | **± 1.3** | **0.03260** | **± 0.00022** | **2533** | **± 101** | **3 484** | **± 25** | **3 478** | **± 25** | **37.0** | **± 1.3** |
| **S1-K** | **153.8** | **785.27** | **± 0.94** | **536.2** | **± 7.7** | **36.4** | **± 1.5** | **0.03412** | **± 0.00016** | **824** | **± 12** | **3 650** | **± 18** | **3 632** | **± 20** | **36.8** | **± 1.5** |
| **S1-L** | **170.7** | **783.41** | **± 0.85** | **95.8** | **± 6.2** | **35.0** | **± 1.2** | **0.03378** | **± 0.00017** | **4556** | **± 294** | **3 618** | **± 19** | **3 615** | **± 19** | **35.4** | **± 1.2** |
| **S1-M** | **179.1** | **941.8** | **± 1.2** | **171.4** | **± 6.3** | **33.7** | **± 1.4** | **0.03569** | **± 0.00017** | **3232** | **± 120** | **3 831** | **± 19** | **3 826** | **± 19** | **34.0** | **± 1.4** |
| **S1-N** | **184.8** | **1007.7** | **± 1.2** | **529.0** | **± 5.4** | **37.4** | **± 1.2** | **0.03458** | **± 0.00017** | **1086** | **± 12** | **3 697** | **± 19** | **3 683** | **± 20** | **37.8** | **± 1.2** |
| **S1-6** | **194.6** | **905.2** | **± 1.4** | **159.7** | **± 5.3** | **33.9** | **± 1.9** | **0.03810** | **± 0.00031** | **3561** | **± 121** | **4 093** | **± 34** | **4 089** | **± 35** | **34.3** | **± 1.9** |
|  |  |  |  |  |  |  |  |  |  |  |  |  |  |  |  |  |  |
| **S1-7** | **200.0** | **899.4** | **± 1.8** | **123.0** | **± 3.1** | **54.5** | **± 3.4** | **0.04065** | **± 0.00029** | **4899** | **± 129** | **4 285** | **± 34** | **4 281** | **± 34** | **55.2** | **± 3.5** |
| **S1-O** | **206.2** | **779.96** | **± 0.82** | **138.8** | **± 6.7** | **43.0** | **± 1.2** | **0.04105** | **± 0.00021** | **3805** | **± 185** | **4 378** | **± 23** | **4 373** | **± 23** | **43.5** | **± 1.3** |
| **S1-P** | **220.2** | **731.33** | **± 0.84** | **434.8** | **± 8.5** | **38.7** | **± 1.4** | **0.04317** | **± 0.00023** | **1197** | **± 24** | **4 628** | **± 26** | **4 613** | **± 28** | **39.2** | **± 1.4** |
| **S1-8** | **230.8** | **766.5** | **± 1.1** | **121.4** | **± 4.0** | **42.4** | **± 2.3** | **0.04346** | **± 0.00022** | **4523** | **± 149** | **4 642** | **± 26** | **4 638** | **± 26** | **43.0** | **± 2.3** |

Analytical errors are 2σ of the mean.

*^a^* [^238^U] = [^235^U] x 137.818 (±0.65‰) (Hiess et al., 2012); δ^234^U = ([^234^U/^238^U]activity - 1) x 1000.

*^b^* δ^234^U_initial_ corrected was calculated based on ^230^Th age (*T* ), i.e., δ^234^U_initial_ = δ^234^U_measured_ *X* e^λ234*T^ , and *T* is corrected age.

*^c^* [^230^Th/^238^U]_activity_ = 1 - e^-λ230^*^T^* + (δ^234^U_measured_/1000)[λ_230_/(λ_230_ - λ_234_)](1 - e^-(λ230 - λ234)^ *^T^* ), where *T* is the age. Decay constants are 9.1705 x 10^-6^ yr^-1^ for ^230^Th, 2.8221 x 10^-6^ yr^-1^ for ^234^U (Cheng et al., 2013), and 1.55125 x 10^-10^ yr^-1^ for ^238^U (Jaffey et al., 1971).

*^d^* Age corrections, relative to chemistry date on February 14th, 2015, were calculated using an estimated atomic 230Th/232Th ratio of 4 (± 2) x 10^-6^. Those are the values for a material at secular equilibrium, with the crustal ^232^Th/^238^U value of 3.8. The errors are arbitrarily assumed to be 50%.

References in S1 Table:

Cheng H, Lawrence Edwards R, Shen C-C, Polyak VJ, Asmerom Y, Woodhead J, et al. Improvements in 230Th dating, 230Th and 234U half-life values, and U–Th isotopic measurements by multi-collector inductively coupled plasma mass spectrometry. Earth Planet Sci Lett. 2013;371-372: 82–91. doi:10.1016/j.epsl.2013.04.006

Hiess J, Condon DJ, McLean N, Noble SR. 238U/235U Systematics in Terrestrial Uranium-Bearing Minerals. Science. 2012;335: 1610–1614. doi:10.1126/science.1215507

Jaffey AH, Flynn KF, Glendenin LE, Bentley WC, Essling AM. Precision Measurement of Half-Lives and Specific Activities of U 235 and U 238. Phys Rev C. 1971;4: 1889–1906. doi:10.1103/PhysRevC.4.1889
