# Supplementary material for: Late Bronze Age climate change and the destruction of the Mycenaean Palace of Nestor at Pylos
Source: PLoS One. 2017 Dec 27;12(12):e0189447. doi: 10.1371/journal.pone.0189447 (PMC5744937; doi:10.1371/journal.pone.0189447)
Supplement: S1 File — (DOCX) [file pone.0189447.s007.docx]

**Archaeological background to the Palace of Nestor at Pylos and the area around Pylos**

The Mycenaean Palace of Nestor at Pylos has produced one of the largest archives of Linear B tablets in the Mycenaean world. The texts themselves are short-term records from the final year of the settlement; they were never meant to be preserved, but were baked as a result of the fire that destroyed the Palace. They are purely administrative in character and speak to the nature of many, although not all, aspects of the Mycenaean economy. The Palace itself has been continuously studied from 1952 when Carl Blegen, from the University of Cincinnati, resumed excavations after the Greek Civil War. From a combination of archaeological and textual evidence, we can extrapolate the catastrophic consequences that climate change would have wrought on the fragile economy of a particular Mycenaean polity.

The Mycenaean civilization reached its zenith in the LH III period and survey data from the area around the Palace at Pylos show a marked increase in the number and size of sites in the vicinity [1,2]. During that period, the extent of the palatial settlement on the ridge doubled from 7 ha at the beginning of the Late Bronze Age to approximately 14-15 ha [3]. Although the last palace was not constructed on the ridge until the final phase of the Late Bronze Age (LH IIIB = around 3280 yrs BP), excavation has revealed evidence of a substantial, earlier structure that probably functioned as a centralized administrative center. Bennet [3] suggests that in the first phase of LH III (LH IIIA = 3370-3280 yrs BP), the Palace of Nestor at Pylos consolidated its supremacy over the surrounding territory and in the final phase of LH IIIB, incorporated the area of eastern Messenia into its polity, bringing the total area controlled by the Palace to ca. 2000 km^2^. Thus, by the middle of the LH IIIB phase, the palace polity consisted of a tenuous political unity that had been cobbled together from independent chiefdoms throughout the Early Mycenaean Period, most probably by means of warfare.

**References**

1. Davis JL, Alcock SE, Bennet J, Lolos YG, Shelmerdine CW. The Pylos Regional Archaeological Project Part I: Overview and the Archaeological Survey. Hesperia. 1997;66: 391. doi:10.2307/148395

2. Davis JL, Bennet J, editors. The Pylos Regional Archaeological Project: A Retrospective. Princeton: American School of Classical Studies at Athens; 2017.

3. Bennet J. The Aegean Bronze Age. In: Scheidel W, Morris I, Saller RP, editors. The Cambridge economic history of the Greco-Roman world. Cambridge, UK ; New York: Cambridge University Press; 2007.
