# Supplementary material for: Late Bronze Age climate change and the destruction of the Mycenaean Palace of Nestor at Pylos
Source: PLoS One. 2017 Dec 27;12(12):e0189447. doi: 10.1371/journal.pone.0189447 (PMC5744937; doi:10.1371/journal.pone.0189447)
Supplement: S2 File — (DOCX) [file pone.0189447.s008.docx]

**Setting**

Mavri Trypa Cave is located 70 m above sea level on a west-facing slope on Schiza Island (approximately 12 km² in size). There is approximately 40 m of bedrock above the cave. The bedded limestones are of Paleocene to Eocene age and belong to the Gavrovo-Tripolitza zone; bituminous material is often present [1,2]. Above the cave, soil cover is thin and patchy and outcrops of the highly karstified limestone bedrock are prevalent. The vegetation consists of thorny shrubs and herbaceous plants along with some smaller trees.

The cave entrance is approximately 4×1.5 m and partly covered by an artificial stonewall. The cave consists of a main passage and two main chambers. During two November visits separated by several years, both occurring before the winter rains had started, occasional dripping was noted throughout the cave, especially in the inner chamber where stalagmite S1 was collected. There is little air movement in the inner chamber and measurements of cave-air relative humidity and temperature showed values of 93% and 19.6°C respectively.

The climate in the area is Mediterranean with hot, dry summers and mild, wet winters. Annual average air temperature at the meteorological station in Methoni (approximately 64 km to the east, elevation 53 m), is 18.0±0.4°C (period 1951-2008) and the annual average precipitation is 698±151 mm (1951-2008). Nearly all precipitation falls between October and April. Evapotranspiration exceeds precipitation in the period from May to September which, in combination with thin soils, restricts infiltration into the bedrock during these months [3]. The prevailing wind direction is west-northwest to south, bringing in moist air from the Mediterranean. The topography of the area promotes orographically induced precipitation to fall on the west-facing hillside.

Schiza Island is uninhabited today and traces of human activities, both past and present, are scarce. Wild goats live on the island and likely originate from previously present domestic animals. No systematic archaeological work has been undertaken in relation to Schiza Island or the Mavri Trypa Cave, although sporadic archaeological finds have been reported from the island and the cave [4,5]. The remote location of Schiza Island, approximately 40 km off the southwestern tip of the Peloponnesian mainland, the rugged terrain of the island and the lack of easy access points, in combination with the thin soil cover and dry climate, suggest that the island may not have been the best candidate for large scale human habitation. Intensive archaeological survey, however, would be needed to investigate in detail the ancient use of the island.

**References**

1. van Hinsbergen DJJ, van der Meer DG, Zachariasse WJ, Meulenkamp JE. Deformation of western Greece during Neogene clockwise rotation and collision with Apulia. Int J Earth Sci. 2006;95: 463–490. doi:10.1007/s00531-005-0047-5

2. Fytrolakis N. Geological map of Greece. Koroni-Pylos-Skhiza sheet. Department of Geological Maps of I.G.M.E; 1980.

3. Genty D, Deflandre G. Drip flow variations under a stalactite of the Père Noël cave (Belgium). Evidence of seasonal variations and air pressure constraints. J Hydrol. 1998;211: 208–232. doi:10.1016/S0022-1694(98)00235-2

4. Valmin N. Malthi-Epilog, Vorläufiger Bericht über die schwedische Ausgrabung in Messenien 1952. Opusc Atheniensia. 1953; 29–46.

5. Davis JL, Alcock SE, Bennet J, Lolos YG, Shelmerdine CW. The Pylos Regional Archaeological Project Part I: Overview and the Archaeological Survey. Hesperia. 1997;66: 391. doi:10.2307/148395
