# Supplementary material for: Late Bronze Age climate change and the destruction of the Mycenaean Palace of Nestor at Pylos
Source: PLoS One. 2017 Dec 27;12(12):e0189447. doi: 10.1371/journal.pone.0189447 (PMC5744937; doi:10.1371/journal.pone.0189447)
Supplement: S3 File — (DOCX) [file pone.0189447.s009.docx]

**Material and Methods**

Stalagmite S1 is ~230 mm long (**FIG 1**). The stalagmite was already detached when collected from the cave floor in the inner chamber approximately 30 meters from the entrance. From the center of the stalagmite, a one-centimeter thick slab was cut parallel to the growth axis. Extraction of samples for stable isotope analysis and uranium thorium dating was done on opposite sides of the thick section. A visual inspection of the central slab shows that the position of the growth axis changed several times during the formation. Petrographic thin sections (30 μm) were produced from a side facing the central slab and analyzed under Nikon Optiphot2-Pol under x25 and x100 magnification.

Twenty four subsamples of 50-100 mg were drilled along growth lamina and mixed with a triple-spike ^229^Th-^233^U-^236^U, which was employed for U and Th fractionation correction. Uranium and thorium separation was conducted in a class-10000 clean room (class-100 bench) following the previous chemistry method [1]. Chemistry procedure was described in Shen et al. [2,3]. Isotopic composition was determined on a multi-collector inductively couple plasma mass spectrometer, Thermo-Finnigan Neptune, at the High-Precision Mass Spectrometry and Environment Change Laboratory (HISPEC), Department of Geosciences, National Taiwan University, Taiwan [4]. Half-lives of U-Th nuclides used for ^230^Th age calculation are given in by Cheng et al. [5]. Isotopic and age errors given are two standard deviation of the mean and two standard deviation.

For stable oxygen and carbon isotope analysis samples of ~0.2 mg were drilled along the growth axis. An overview of stalagmite S1 was done at 1-2 mm-resolution using a hand-held drill fitted with a diamond drill bit. This was followed by sub-millimeter micromilling in slower growing sections of the stalagmite, i.e. the major part of the stalagmite, in order to obtain an even temporal resolution. In total 355 samples were analyzed along the growth axis of the stalagmite.

For stable isotope analysis 0.2 mg carbonate was flushed with argon gas in a septum-seal glass vial. 100 μl of 99 % H_3_PO_4_ was added to each sample for reacting to carbon dioxide. Analyses were conducted using a GasbenchII coupled to a Finnigan MAT 252 mass spectrometer at the stable isotope laboratory of Friedrich-Alexander University, Erlangen-Nürnberg. Reproducibility and accuracy were monitored by replicate analysis of laboratory standards calibrated to NBS19 and LSVEC. One-sigma precision is better than ±0.06 ‰ for δ^13^C and ±0.10 ‰ for δ^18^O.

**References**

1. Edwards RL, Chen JH, Wasserburg GJ. 238U234U230Th232Th systematics and the precise measurement of time over the past 500,000 years. Earth Planet Sci Lett. 1987;81: 175–192. doi:10.1016/0012-821X(87)90154-3

2. Shen C-C, Lawrence E, Cheng H, Dorale JA, Thomas RB, Bradley M, et al. Uranium and thorium isotopic and concentration measurements by magnetic sector inductively coupled plasma mass spectrometry. Chem Geol. 2002;185: 165–178. doi:10.1016/S0009-2541(01)00404-1

3. Shen C-C, Cheng H, Edwards RL, Moran SB, Edmonds HN, Hoff JA, et al. Measurement of Attogram Quantities of ^231^ Pa in Dissolved and Particulate Fractions of Seawater by Isotope Dilution Thermal Ionization Mass Spectroscopy. Anal Chem. 2003;75: 1075–1079. doi:10.1021/ac026247r

4. Shen C-C, Wu C-C, Cheng H, Lawrence Edwards R, Hsieh Y-T, Gallet S, et al. High-precision and high-resolution carbonate 230Th dating by MC-ICP-MS with SEM protocols. Geochim Cosmochim Acta. 2012;99: 71–86. doi:10.1016/j.gca.2012.09.018

5. Cheng H, Lawrence Edwards R, Shen C-C, Polyak VJ, Asmerom Y, Woodhead J, et al. Improvements in 230Th dating, 230Th and 234U half-life values, and U–Th isotopic measurements by multi-collector inductively coupled plasma mass spectrometry. Earth Planet Sci Lett. 2013;371-372: 82–91. doi:10.1016/j.epsl.2013.04.006
