# Supplementary material for: Late Bronze Age climate change and the destruction of the Mycenaean Palace of Nestor at Pylos
Source: PLoS One. 2017 Dec 27;12(12):e0189447. doi: 10.1371/journal.pone.0189447 (PMC5744937; doi:10.1371/journal.pone.0189447)
Supplement: S4 File — (DOCX) [file pone.0189447.s010.docx]

**Results**

**Petrography**

Stalagmite S1 primarily consists of open columnar calcite throughout the sample. The occurrence of micrite and new crystal nucleation indicate growth interruptions at 47.7 mm and 197.5 mm depth from the top of the stalagmite (**FIG 1 and S1 FIG**). Irregular and fibrous appearance of the fabric, indicative of dissolutional processes, are apparent in the central area of the stalagmite between 55 mm and 145 mm depth. This area was avoided when sampling for stable isotopes and U-Th dating.

**U-Th dating**

The corrected U-Th ages for stalagmite S1 are precise with an average uncertainty <±1 % (maximum uncertainty is ±3 % occurring in one sample) (**FIG 3 and S1 Table**). Nearly all U-Th ages, within uncertainties, fall in stratigraphical order (**FIG 3**). Bedrock properties above Mavri Trypa contribute to a relatively high uranium content; the average [^238^U] is 901.85 ppb. Low levels of detrital thorium are indicated by high [^230^Th/^232^Th] values; the average is 3415 ppm. This means that only minimal corrections are needed [1].

Large jumps in ages between adjacent U-Th samples with low uncertainties are found in association with micritic fabrics identified in petrographic thin sections. This confirms that micritic fabrics are associated with extended hiatuses.

**Age-depth model**

Age-depth modeling is based on the corrected U-Th ages and information from petrographic analysis. *StalAge* (v. 1.0) [2] was used to produce three separate age-depth models for the three discrete growth periods (#1-3) indicated both by U-Th ages and petrographic analysis. The combined age-depth model suggests that stalagmite S1 formed between 4687±68 and 1297±103 yrs BP with interruptions in growth between 4182±33 and 3813±370 yrs BP and between 2953±63 and 2067±27 yrs BP (**FIG 3**). The three individual growth periods extend from 4687±68 to 4182±33 yrs BP (#1), from 3813±370 to 2953±63 yrs BP (#2) and from 2067±27 to 1297±103 yrs BP (#3) (**FIG 3**). Following the age-depth model, the temporal resolution of the stable isotope samples is on average 6 years per sample.

Age uncertainties are generally small and the age-model building assumes ages are accurate. Modelled ages presented in this paper are identified by their median StalAge age estimate and the age uncertainties should be acknowledged for interpretations and comparisons.

**Stable isotopes**

The range of δ^18^O and δ^13^C along the growth axis is from -3.74 to -5.99 ‰ (V-PDB) and from -6.15 to -11.07 ‰ (V-PDB), respectively (**S2 FIG**). The correlation between δ^18^O and δ^13^C along the growth axis in the three growth periods was investigated using Pearson product-moment correlation. There is a positive correlation between δ^18^O and δ^13^C in all three growth periods: #3 r=0.66 (n=96), #2 r=0.84 (n=146), and #1 r=0.80 (n=113) all significant at the 95 % level. The stable isotope signal is stable in the stalagmite, as illustrated by the close similarity of the isotope results from parallel tracks (**S5 FIG**)

**References**

1. Hellstrom J. U–Th dating of speleothems with high initial 230Th using stratigraphical constraint. Quat Geochronol. 2006;1: 289–295. doi:10.1016/j.quageo.2007.01.004

2. Scholz D, Hoffmann DL. StalAge – An algorithm designed for construction of speleothem age models. Quat Geochronol. 2011;6: 369–382. doi:10.1016/j.quageo.2011.02.002
