# Supplementary material for: Late Bronze Age climate change and the destruction of the Mycenaean Palace of Nestor at Pylos
Source: PLoS One. 2017 Dec 27;12(12):e0189447. doi: 10.1371/journal.pone.0189447 (PMC5744937; doi:10.1371/journal.pone.0189447)
Supplement: S5 File — (DOCX) [file pone.0189447.s011.docx]

**Extended discussion: Potential impacts of climate variability on Mycenaean society**

Because the Mycenaean economy was centered around agriculture, crop-failure was always a risk. Risk coping strategies on a local level, however, were undermined by several factors e.g., the palatial and elite control of vast tracts of land, the taxation of surplus grain from local farmers not under direct elite control and palatial control over certain types of craft production [1]. Since the Palace extracted taxes from most elements of the population, it had obligations to its dependents and the wider community. The Palace would have been expected to provided subsistence relief in times of crisis, an obligation it would have been unable to meet during a series of crisis years. The Linear B texts indicate that the Palace also provided staple and luxury offerings to sanctuaries and supported communal feasting and, presumably, supported the military. Crop failure would have had a severe impact on its ability to meet these obligations.

Prior to the final destruction of the Palace of Nestor at Pylos, the reaction of the palatial elite to economic stress is visible in a move to restrict access to and within the central building, an increase in storage facilities (Wine Magazine and Room 27), the transfer of the production of exchange goods, such as perfumed oil, under the direct control of the administration into the Palace itself and an increased emphasis on control mechanisms such as communal feasting. The latter is evidenced by the large store of unused vessels in the Pantries, and perhaps by even large-scale sacrifices like the one that took place shortly before the destruction of the Palace [2]. These were possible responses to the difficulties created by food shortages, which themselves probably sparked unrest within the predominately agricultural community, especially if the Palace was unable to provide food relief.

**References**

1. Halstead P. Toward a model of Mycenaean palatial mobilization. In: Galaty ML, Parkinson WA, editors. Rethinking Mycenaean palaces II. Rev. and expanded 2nd ed. Los Angeles: Cotsen Institute of Archaeology, University of California; 2007. pp. 66–73.

2. Stocker SR, Davis JL. Animal Sacrifice, Archives, and Feasting at the Palace of Nestor. Hesperia J Am Sch Class Stud Athens. 2004;73: 179–195.
